# Hepatitis E Virus Infection in Iranian Kidney-Transplant Patients

**DOI:** 10.5812/kowsar.1735143X.791

**Published:** 2011-11-30

**Authors:** Nassim Kamar

**Affiliations:** 1 Department of Nephrology, Dialysis and Organ Transplantation, CHU Rangueil, Toulouse, France

**Keywords:** Hepatitis E, Kidney Transplantation, Patients

Dear Editor,

Hepatitis E virus infection is an endemic disease in developing and industrialized countries [1],and is responsible for acute and chronic hepatitis. Genotype 1 is more prevalent in developing countries, whereas genotype 3 is more common in developed countries [1]. Chronic genotype 3 HEV infection can occur in solid-organ transplant patients [2], hematological patients who receive chemotherapy [3], and HIV-positive patients [4]. In the past several years, HEV infection in organ transplant patients has garnered much interest. After kidney transplantation, HEV-related liver fibrosis can lead rapidly to cirrhosis [5]. The use of tacrolimus, rather than cyclosporine A, and a low platelet count at HEV diagnosis have been identified as predictive factors for chronic HEV infection [6]. However, decreased immunosuppressant dose can result in HEV clearance in nearly one-third of patients [6]. In addition, ribavirin monotherapy can be efficacious in treating chronic HEV infection [7].

In this issue of Hepatitis Monthly, Khameneh et al. determined the prevalence of anti-HEV IgG in 91 randomly selected Iranian kidney transplant patients [8]. The HEV seroprevalence was 30.8%, although there was poor sensitivity and specificity between HEV serological assays [9]. However, this study on seroprevalence in an Iranian kidney transplant population is a first step toward improving the assessment of HEV infection in this setting. Unfortunately, as discussed by the authors, HEV RNA was not measured in this study. Consequently, the authors were unable to determine whether some patients developed chronic hepatitis—a significant shortcoming, because 45.1% of patients had unexplained increases in liver function tests [8]. The measurement of HEV genotype may also be valuable, because, similar to what has been observed in Europe, Khameneh et al. found more positive HEV serologies in older patients [8].

In summary, Khamaneh et al. noted a high seroprevalence rate of HEV in an Iranian kidney transplant population. Further studies are needed to evaluate the impact of HEV infection in this population.
